# Effect of α-tocopheryloxy acetic acid, a vitamin E derivative mitocan, on the experimental infection of mice with *Plasmodium yoelii*

**DOI:** 10.1186/s12936-021-03817-9

**Published:** 2021-06-24

**Authors:** Kasumi Kawamura, Aiko Kume, Rika Umemiya-Shirafuji, Shunji Kasai, Hiroshi Suzuki

**Affiliations:** National Research Center for Protozoan Diseases, Obihiro University of Agriculture and Veterinary Medicine, Nishi 2-13, Inada-cho, Obihiro, Hokkaido 080-8555 Japan

**Keywords:** α-tocopheryloxy acetic acid, Mice, *Plasmodium yoelii*, Reactive oxygen species

## Abstract

**Background:**

Malaria parasites are known to be vulnerable to oxidative stress. In this study, the effects of the administration of α-tocopheryloxy acetic acid (α-TEA), which is a vitamin E analogue mitocan, on *Plasmodium yoelii* infection in mice were examined.

**Methods:**

Alpha-TEA was mixed with diet and fed to C57BL/6J mice before and/or after infection. For parasite infection, 4 × 10^4^ red blood cells infected with *P. yoelii* (strain 17XL) were inoculated by intraperitoneal injection. In another series of experiment, the effect of the oral administration of α-TEA on *P. yoelii* 17XL infection in mice was examined. Finally, the combined effect of α-TEA and dihydroartemisinin or chloroquine on *P. yoelii* 17XL infection was examined.

**Results:**

When 0.25% α-TEA was mixed with the diet for 7 days before infection and 14 days after infection (in total for 21 days), for 14 days after infection, and for 11 days from the third day after infection, all *P. yoelii* 17XL-infected mice survived during the observation period. However, all control mice died within 12 days after infection. These results indicated that α-TEA functions effectively even when administered post-infection. The oral administration of α-TEA for *P. yoelii* 17XL infection was also significant. Although the infected mice in the solvent control died within 10 days after infection, 90% of the mice infected with *P. yoelii* 17XL survived during the observation period when treated with 10 mg/head/day of α-TEA for 3 days from day 3 after infection. Although the combined effect of α-TEA and dihydroartemisinin (DHA) or chloroquine on *P. yoelii* 17XL infection was significant, no synergistic or additive effects were observed from the survival curve.

**Conclusions:**

This study showed the beneficial effects of α-TEA on the experimental infection of mice with *P. yoelii* 17XL. The stimulatory action of α-TEA on mitochondria and the accompanying reactions, such as reactive oxygen species production, and induction of apoptosis might have some effect on malarial infection.

## Background

Malaria is a protozoan infection with fever, anaemia, and splenomegaly as the main symptoms. As one of the three major global infectious diseases, about half of the world's population is at risk of malaria. In 2019, there were 229 million cases and 409,000 deaths from malaria. In particular, children under the age of five are most susceptible to malaria, accounting for two-thirds of malaria deaths worldwide [1].

Protozoa, including this malaria parasite, are vulnerable to oxidative stress, and reactive oxygen species (ROS) intermediate the haemolysis of host erythrocytes, endothelial damage, and parasite death in malaria [2, 3]. Vitamin E is a fat-soluble antioxidant and its derivatives produce ROS and exhibits anticancer activity [4, 5]. Alpha-tocopheryl succinate (α-TOS), a derivative of vitamin E, showed anticancer activity against HER2-positive breast cancer in mice [6, 7]. For malaria infection, administration of 50–100 mM of α-TOS on days 1, 3, 5, and 7 after infection with *Plasmodium yoelii* (strain 17XL) or *Plasmodium berghei* (strain ANKA) resulted in a significant increase in host survival in mice [8]. However, since this chemical compound is decomposed by an esterase it has low stability and clinical application may be challenging. Therefore, in this study, the effects of α-tocopheryloxy acetic acid (α-TEA), which is a more stable vitamin E derivative [9], and concomitant use with existing drugs on *P. yoelii* infection in mice were examined. Alpha-TEA is a vitamin E derivative derived and synthesized from α-tocopherol that has an ether bond and is not decomposed by esterase, thus, it can be taken orally [10, 11].

## Methods

C57BL/6 J mice were purchased from Japan CLEA (Tokyo, Japan) and bred in a specific pathogen-free facility. Experimental infection with *P. yoelii* was performed using male mice at a biosafety level 2 facility. The room temperature (24 ± 1 °C) and humidity (50 ± 10%) were regulated, and lighting was controlled (lights on from 7:00 to 19:00). Mice had free access to water and a commercial regular diet (CA-1; CLEA Japan, Tokyo, Japan). The animals used in this study were treated and cared for based on the Guiding Principles for the Care and Use of Research Animals established by Obihiro University of Agriculture and Veterinary Medicine. All animal experimental protocols were approved by the Institutional Animal Ethics Committee, Obihiro University of Agriculture and Veterinary Medicine (Approval number #20–125).

Alpha-TEA distributed by Eisai (Eisai Co., Ltd., Tokyo, Japan) were mixed with diet (0.083% and 0.25% (w/w) of α-TEA) and fed to C57BL/6 J male mice over 8 weeks of age (23–25 g body weight) for 7 days before infection and 14 days after infection (in total for 21 days), for 14 days after infection, and for 11 days from day 3 after infection. Hahn et al. examined the antitumor effect of dietary administration of α-TEA in mice, and showed that α-TEA had a significant dose-dependent antitumor effect at a dose of 0.05 to 0.3% [12]. With reference to this report, 0.083% (low dose) and 0.25% α-TEA diet (high dose) groups were provided in this study. In order to examine the effect of prophylactic administration of the drug, an experimental group was provided as pre- and post-infection administration. The preparation of the mixed-diet was outsourced to CLEA Japan. For preparation of parasites, mice were intraperitoneally administered with 200 μL of cryopreserved infected erythrocytes after thawing at 37° C in water bath. When parasitaemia of the mice exceeded to 10%, 40 μL of blood was collected and mixed with 400 μL of PBS, and of which 200 μL was intraperitoneally administered to mice. Then, blood was collected when the parasitaemia exceeded to 10% and mixed with PBS to adjust the concentration of infected erythrocytes to 2 × 10^5^/mL [8]. For parasite infection, 4 × 10^4^ *P. yoelii* 17XL-infected red blood cells (iRBCs) were inoculated by intraperitoneal injection in α-TEA-treated mice and control mice fed a normal diet, and their parasitaemia and survival rates were monitored. The day of infection was defined as day 0. From day 3 after infection, 2 μL of peripheral blood was collected from the tip of the tail, smeared on a glass slide, stained with Giemsa (Sigma-Aldrich, Tokyo, Japan), and the proportion of infected erythrocytes in total erythrocytes (parasitaemia) was determined using a phase-contrast microscope (DIAPHOTO-TMD300, Nikon, Tokyo, Japan). Over 1,000 erythrocytes were counted in each mouse to assess parasitaemia.

In another series of experiment, the effect of the oral administration of α-TEA on *P. yoelii* 17XL infection in mice was examined. Alpha-TEA was dissolved with 20% DMSO (Dimethyl sulfoxide, Sigma-Aldrich, Tokyo, Japan) in PEG-300 (Polyethylene Glycol 300, Wako, Osaka, Japan). As a preliminary experiment, the pharmacokinetics of single and repeated oral administration of α-TEA was observed, and no abnormalities were observed in the general condition, body weight transition, and liver function markers of the mice under any of the administration conditions. Male mice were infected with 4 × 10^4^ *P. yoelii* 17XL iRBCs and orally administered 0, 1, 3, and 10 mg/head/day of α-TEA from days 3 to 5 after infection, and their parasitaemia and survival rate were monitored.

Finally, the combined effect of α-TEA and dihydroartemisinin (DHA) or chloroquine on *P. yoelii* 17XL infection was examined. Male mice were infected with 4 × 10^4^ *P. yoelii* 17XL iRBCs and orally administered 3 mg/head/day of α-TEA and intraperitoneally injected with 1 mg/kg/day of DHA (D3793, Tokyo Chemical Industry Co., Ltd., Tokyo, Japan) or 1 mg/kg/day of chloroquine (C6628, Sigma-Aldrich Japan, Tokyo, Japan) from days 3 to 5 after infection. When the combined effect of the two compounds was examined, in order to clarify the combined effect of the two compounds, the doses at which a survival rate of about 50% was observed when each drug was administered alone were used [13, 14].

As the control, a solvent control group (0 mg α-TEA) in which the parasite and the solvent were administered and a control group in which only the parasite was administered were provided. Each infectious experiment was repeated at least three times and showed typical and representative results. Parasitaemia was analysed using the One-way ANOVA and Tukey test. Survival rate was analysed using the log-rank (Mantel-Cox) and Gehan-Breslow-Wilcoxon tests implemented in GraphPad Prism 5. For all analyses, a value of p < 0.05 was considered statistically significant.

## Results

**Fig. 1:**
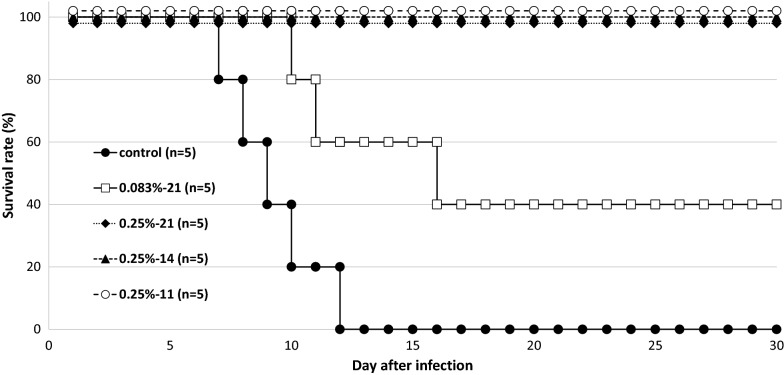
Effect of α-TEA mixed with diet on survival after *P. yoelii* 17XL infection in mice. In total, 0.083% and 0.25% (w/w) α-TEA were mixed with diet and fed to C57BL/6J mice for seven days before 4 × 10^4^ iRBCs and 14 days after infection (0.083%-21, 0.25%-21), for 14 days after infection (0.25%-14), and for 11 days from the third day after infection (0.25%-11). Control vs. 0.083%-21; P < 0.05. Control vs. 0.25%-21, -14, and -11; P < 0.05. 0.083%-21 vs. 0.25%-21, -14, and -11; P = 0.05

**Fig. 2:**
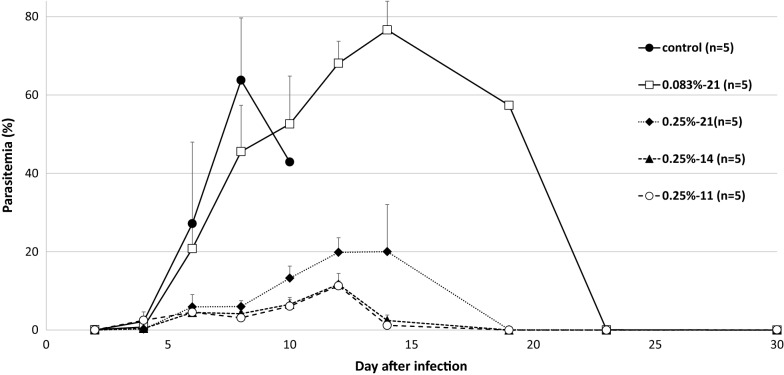
Effect of α-TEA mixed with diet on parasitaemia after *P. yoelii* 17XL infection in mice. In total, 0.083% and 0.25% (w/w) α-TEA were mixed with diet and fed to C57BL/6 J mice for 7 days before 4 × 10^4^ iRBCs and 14 days after the infection (0.083%-21, 0.25%-21), for 14 days after infection (0.25%-14), and for 11 days from day 3 after infection (0.25%-11). Error bars indicate standard error. Control vs. 0.25%-21, -14, and -11 on day 8; P < 0.05. 0.083%-21 vs. 0.25%-21 on days 4, 6, 8, 12, 14, and 19; P < 0.05. 0.083%-21 vs. 0.25%-14 on days 6 and 8, 10, 12, 14, and 19; P < 0.05. 0.083%-21 vs. 0.25%-14 on days 8, 10, 12, and 14; P < 0.05. 0.25%-21 vs. 0.25%-11 on days 4, 8, 10, 12, and 14; P < 0.05. 0.25%-14 vs. 0.25%-11 on day 4; P < 0.05

As shown in Fig. 1, the effect of α-TEA mixed with diet on *P. yoelii* 17XL infection was significant. When 0.25% α-TEA was mixed with the diet for 7 days before infection and 14 days after infection (in total for 21 days), for 14 days after infection, and for 11 days from the third day after infection, all *P. yoelii* 17XL-infected mice survived during the observation period. However, all control mice died within 12 days after infection. Although the effect of lower concentrations of α-TEA (0.083%) was limited, the survival rate remained significantly higher than that of the control group (P < 0.05). Regarding parasitaemia (Fig. 2), the control and 0.083% groups showed almost the same changes in proportions of parasitaemia after infection, and there was no significant difference between the two groups (P > 0.05). When 0.25% α-TEA was administered for 7 days before infection and 14 days after infection (in total for 21 days), proportions of parasitaemia were significantly higher than when 0.25% α-TEA was administered for 14 days after infection on days 8, 10, 12, and 14 after infection (P < 0.05), and for 11 days after infection on days 4, 8, 10, 12, and 14 after infection (P < 0.05). Therefore, it was clarified that α-TEA functions effectively even when administered post-infection. And the prophylactic effect of α-TEA on parasitaemia may be limited.

**Fig. 3:**
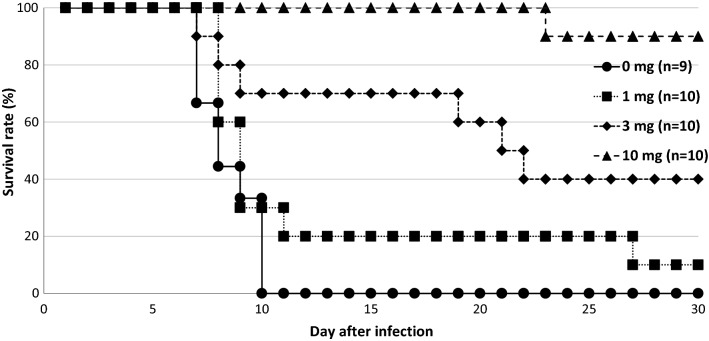
Effect of oral administration of α-TEA on survival after *P. yoelii* 17XL infection in mice. C57BL/6 J mice infected with 4 × 10^4^ iRBCs were orally administered 0, 1, 3, and 10 mg/head/day of α-TEA for 3 days from day 3 after infection. As the control, a solvent control group (0 mg α-TEA) in which the parasite and the solvent were administered was provided. 10 mg/head/day vs. 0, 1, and 3 mg/head/day; P < 0.05. 3 mg/head/day vs. 0 mg/head/day; P < 0.05

**Fig. 4:**
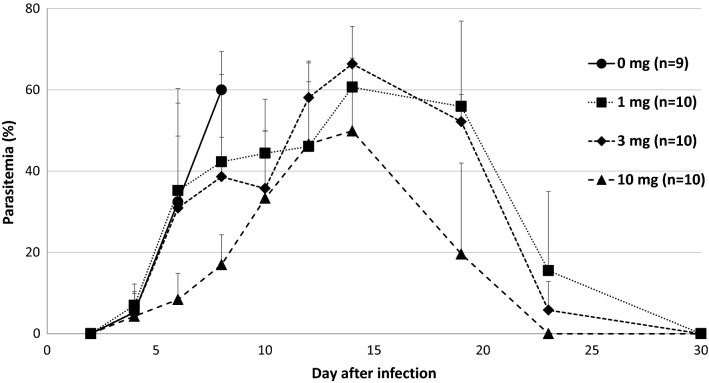
Effect of the oral administration of α-TEA on parasitaemia after *P. yoelii* 17XL infection in mice. C57BL/6 J mice infected with 4 × 10^4^ iRBCs were orally administered 0, 1, 3, and 10 mg/head/day of α-TEA for 3 days from day 3 after infection. As the control, a solvent control group (0 mg α-TEA) in which the parasite and the solvent were administered was provided. Error bars indicate standard error. 0 mg/head/day vs. 3 mg/head/day on day 6; P < 0.05. 0 mg/head/day vs. 10 mg/head/day on days 6 and 8; P < 0.05. 1 mg/head/day vs. 10 mg/head/day on days 6, 8, and 19; P < 0.05. 3 mg/head/day vs. 10 mg/head/day on days 6, 8, 14, and 19; P < 0.05

As shown in Fig. 3, the oral administration of α-TEA for *P. yoelii* 17XL infection was also significant. Although the infected mice in the solvent control (0 mg α-TEA) died within 10 days after infection, 90% of the mice infected with *P. yoelii* 17XL survived during the observation period when treated with 10 mg/head/day of α-TEA for 3 days from day 3 after infection. The survival rates of the 10 mg/head/day group were significantly higher than those in the 1 and 3 mg/head/day groups (P < 0.05). The survival rates of the 3 and 10 mg/head/day groups were significantly higher than that of the solvent control group (P < 0.05). When the infected mice were treated with α-TEA for 3 days from day 5 after infection, the survival rate decreased to about half that of the treatment from the third day. For parasitaemia (Fig. 4), although all experimental groups showed an increase in the proportion of parasitaemia after infection, the proportions in the 3 and 10 mg/head/day groups on day 6 after infection were significantly lower than in the solvent control group (P < 0.05). In addition, the proportion of parasitaemia in the 10 mg/head/day group was significantly lower than those in the 1 and 3 mg/head/day groups on days 6, 8, and 19 after infection (P < 0.05).

**Fig. 5:**
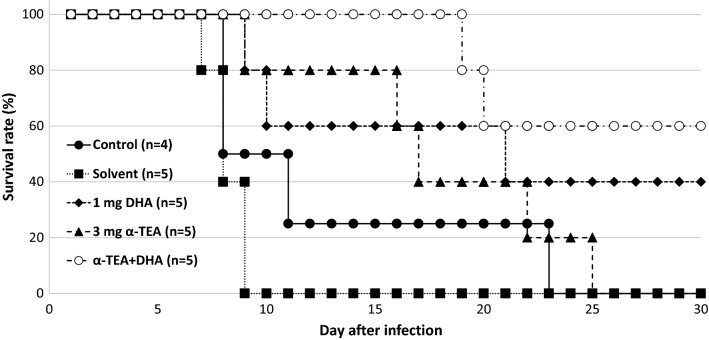
The combined effect of α-TEA and DHA on survival after *P. yoelii* 17XL infection in mice. C57BL/6J mice infected with 4 × 10^4^ iRBCs were administered 3 mg/head/day of α-TEA and 1 mg/kg/day DHA for 3 days from day 3 after infection. As the control, a solvent control group (0 mg α-TEA) in which the parasite and the solvent were administered and a control group in which only the parasite was administered were provided. Control and solvent vs. α-TEA + DHA; P < 0.05. Solvent vs. DHA and α-TEA; P < 0.05. α-TEA vs. α-TEA + DHA; P = 0.06

**Fig. 6:**
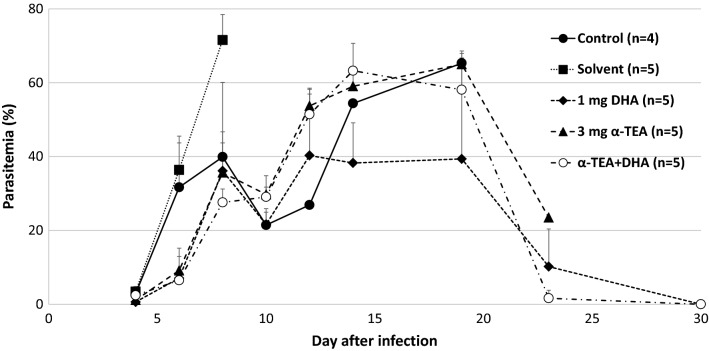
The combined effect of α-TEA and DHA on parasitaemia after *P. yoelii* 17XL infection in mice. C57BL/6J mice infected with 4 × 10^4^ iRBCs were administered 3 mg/head/day of α-TEA and 1 mg/kg/day DHA for 3 days from day 3 after infection. As the control, a solvent control group (0 mg α-TEA) in which the parasite and the solvent were administered and a control group in which only the parasite was administered were provided. Error bars indicate standard error. DHA vs. α-TEA + DHA on day 4; P < 0.05

Figure 5 shows the combined effect of α-TEA and DHA on survival after *P. yoelii* 17XL infection in mice. There was no significant difference in survival rate after infection between the solvent and untreated controls. All solvent control mice and non-treated control mice died by day 9 and 23 post-infection, respectively. When mice were administered both α-TEA (3 mg/head/day) and DHA (1 mg/kg/day) for 3 days from day 3 after infection, they showed higher survival compared with both the solvent and non-treated control groups (P < 0.05), but not the α-TEA (P = 0.06) and DHA (P > 0.05) groups. As shown in Fig. 6, parasitaemia remained similar in all experimental groups, but there was a significant difference between DHA and the combination group on day 4 (P < 0.05).

**Fig. 7:**
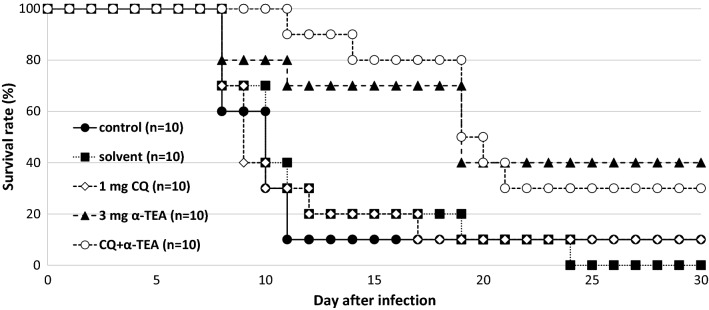
The combined effect of α-TEA and chloroquine on survival after *P. yoelii* 17XL infection in mice. C57BL/6J mice infected with 4 × 10^4^ iRBCs were administered 3 mg/head/day of α-TEA and 1 mg/kg/day chloroquine for 3 days from day 3 after infection. As the control, a solvent control group (0 mg α-TEA) in which the parasite and the solvent were administered and a control group in which only the parasite was administered were provided. Control, solvent, and chloroquine vs. α-TEA + chloroquine; P < 0.05. Control vs. α-TEA; P = 0.05. Solvent vs. α-TEA; P < 0.05. α-TEA vs. chloroquine; P < 0.05

**Fig. 8:**
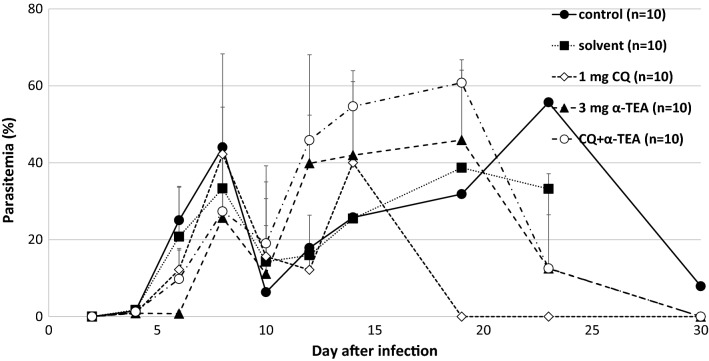
The combined effect of α-TEA and chloroquine on parasitaemia after *P. yoelii* 17XL infection in mice. C57BL/6J mice infected with 4 × 10^4^ iRBCs were administered 3 mg/head/day of α-TEA and 1 mg/kg/day chloroquine for 3 days from day 3 after infection. As the control, a solvent control group (0 mg α-TEA) in which the parasite and the solvent were administered and a control group in which only the parasite was administered were provided. Error bars indicate standard error. Control vs. α-TEA + chloroquine on days 6, 10, and 19; P < 0.05. Control vs. α-TEA on day 8; P < 0.05

As shown in Fig. 7, although the combined effect of α-TEA and chloroquine on *P. yoelii* 17XL infection was significant, no synergistic or additive effects were observed from the survival curve. Namely, survival after the combined administration of α-TEA (3 mg/head/day) and chloroquine (1 mg/kg/day) for 3 days from day 3 after infection was significantly higher than that in both controls and chloroquine (P < 0.05), but not in the single administration of α-TEA (P > 0.05). Chloroquine was used at sub-curative dose, so its effect was limited on the survival curve. In the transition of parasitaemia (Fig. 8), although the combination of α-TEA and chloroquine tended to be similar to the α-TEA and chloroquine alone groups. The proportions of parasitaemia in the α-TEA and chloroquine combination groups were significantly lower than that in the control group on day 6 after infection (P < 0.05).

## Discussion

This study showed the beneficial effects of α-TEA on the experimental infection of mice with *P. yoelii* 17XL. There have been some studies regarding the anti-tumour effects of α-TEA [15–21], but the anti-pathogen or anti-protozoal effects have not yet been investigated. When *P. yoelii* 17XL-infected mice were treated with α-TEA via being mixed into the diet or through oral administration, their viability was almost completely maintained (Figs. 1 and 3) and the parasites were eliminated from their blood (Figs. 2 and 4). However, the effect of pretreatment with α-TEA on parasitaemia was limited. Specifically, there was no difference in survival rates whether administration commenced before or after infection, although there was a difference in the transition of parasitaemia. When comparing the groups that received α-TEA either before or after infection, the latter significantly reduced parasitaemia on days 8, 10, 12, 14, and 19 after infection (Fig. 2, P < 0.05). These data indicate that it may be possible to maintain parasitaemia lower by increasing the blood α-TEA concentration just before the elevation of parasitaemia after malarial infection. Furthermore, α-TEA may act directly on the protozoa rather than modifying the host environment. It has been reported that the administration of 50–100 mM of α-TOS after *P. yoelii* 17XL infection significantly increases host survival in mice [8]. However, all α-TOS-treated mice died within 20 days of infection, while α-TEA administration rescued almost all infected mice in this study (Figs. 1 and 3). As α-TEA is not hydrolysed and has good stability [10, 11], it may be an effective anti-malarial drug candidate. In our preliminary pharmacokinetic experiment, in which 0.25% α-TEA-mixed diet was fed to uninfected C57BL/6 J mice for 21 days, the average food intake of the mice was 3.6 g/head/day. The daily food intake was comparable to mice fed with a normal chew diet. From this value the average α-TEA intake of mice is 9 mg/head/day. The elimination half-life (T_1/2_) was 61.3 h after a single oral dose of α-TEA (10 mg/head) in mice; the maximum concentration (C_max_) was 25.7 μg/mL and the area under the blood concentration time curve (AUC) was 1780 μg·h/mL. In addition, the mice orally administered with α-TEA (10 mg/day) for 3 days had a plasma α-TEA concentration of 10 μg/mL or higher for 6 days from the start of administration (Kasai et al., unpublished data).

When DHA was combined with α-TEA for *P. yoelii* 17XL infection, no synergistic effect was observed. However, α-TEA and DHA did not antagonize, and there may have been an additive effect (Fig. 5). The survival rate on day 30 was 0% in the 3 mg α-TEA and 40% in the 1 mg DHA group, whereas this was 60% in the DHA (1 mg) with α-TEA (3 mg) group. The survival rate by day 30 in the DHA with α-TEA group was significantly higher than that in the control group (P < 0.05). Although there was no difference between the DHA with α-TEA group and DHA (P > 0.05) and α-TEA group (P = 0.06), the survival rate tended to increase in the combined group. On the other hand, the combined use of α-TEA and chloroquine in *P. yoelii-*infected mice did not have a clear combined effect on their survival rate (Fig. 7). In the combined group, parasitaemia increased sharply after the end of the administration period (Fig. 8), suggesting that the combination effect may be exerted more strongly by extending the administration period.

It remains unclear whether anti-malarial effects of α-TEA were caused by impact on parasites directly, regulation of pathological mechanisms or enhancement of host defense mechanisms such as the immune system. However, parasites might certainly intake α-TEA from plasma and erythrocyte membrane. In our preliminary experiment, the number of trypanosome was significantly lower following α-TEA treatment than that after control treatment (no α-TEA) in culture in vitro, suggesting that α-TEA directly affected trypanosome. α-TEA induced the production of ROS, such as hydroxyl radical and peroxynitrite, in protozoans. (Kawamura et al., unpublished observation). Oxidative stress may inhibit the development of parasite growth. Alpha-TEA stimulates mitochondria to produce ROS and induces the apoptosis of tumor cells [16, 20, 22] as well as enhances the anti-tumour activity of trastuzumab against HER2/neu-expressing breast cancer [17, 18]. As α-TEA had a stronger inhibitory effect on breast cancer than α-TOS, and is more stable in plasma [9], it is expected that the stimulatory action of α-TEA on mitochondria [23] and the accompanying reactions, such as ROS production, induction of apoptosis, and stimulation of autophagy [19] might have some effect on malarial infection. Parasites exposed to α-TEA would readily accumulate ROS by interfering with the mitochondrial redox chain and activating the intrinsic apoptotic pathway. Endothelial cells deficient in mitochondrial DNA are resistant to α-TEA, both in ROS accumulation and apoptosis induction, maintaining their angiogenic potential [24]. Furthermore, α-TEA is a potent anti-tumor agent with a safe toxicity profile in mice [25] and dogs [26].

## Conclusion

In this study, α-TEA was effective against murine malaria, but no combination effect with DHA or chloroquine was observed. Since a sufficient anti-malarial effect can be obtained with α-TEA alone, future studies could focus on the combined effect of α-TEA with other existing drugs and consider a drug with a more combined effect. The prophylactic anti-malarial activity of premedication with α-TEA may also be interesting. Furthermore, it is expected that this compound will be developed as an antiprotozoal drug by analysing the mechanism of action of α-TEA and examining its effect on other protozoan infections in future.

## Data Availability

The data that support the findings of this study are available from the corresponding author upon reasonable request.
